# Clinical Characterization of a National Cohort of Patients With Germline *WT1* Variants Including Late-Onset Phenotypes

**DOI:** 10.1016/j.ekir.2024.09.007

**Published:** 2024-09-16

**Authors:** Sophie E. van Peer, Roland P. Kuiper, Janna A. Hol, Sanne Egging, Bert van der Zwaag, Marc R. Lilien, M. Paola Lombardi, Marry M. van den Heuvel-Eibrink, Marjolijn C.J. Jongmans

**Affiliations:** 1 Princess Máxima Center for Pediatric Oncology, Utrecht, the Netherlands; 2 Department of Clinical Genetics, Erasmus MC, Rotterdam, the Netherlands; 3 Department of Genetics, University Medical Center Utrecht, Utrecht, the Netherlands; 4 Department of Pediatric Nephrology, Wilhelmina Children's Hospital/University Medical Center Utrecht, Utrecht, the Netherlands; 5 Department of Human Genetics, Laboratory for Genome Diagnostics, Amsterdam UMC, Amsterdam, the Netherlands; 6 Division of Child Health, Wilhelmina Children's Hospital/University Medical Center Utrecht, Utrecht, the Netherlands

**Keywords:** Denys-Drash, Wilms tumor, WT1

## Abstract

**Introduction:**

*WT1* disorder is a recently introduced term for phenotypes associated with germline Wilms Tumor 1 (*WT1*) variants, including glomerulopathy, urogenital anomalies, and Wilms tumor. Previous studies showed a bias toward missense variants in the DNA-binding/Zinc-finger domain of *WT1* (exon 8 and 9) and patients with early-onset glomerulopathy. Thorough genotype-phenotype correlations including follow-up data on late-onset glomerulopathy risk are lacking. To characterize the genotypic and phenotypic spectrum of *WT1* disorder, we describe a national cohort of individuals with *WT1* variants.

**Methods:**

We requested clinical and genetic data of all patients with germline *WT1* variants at all Dutch genetic laboratories.

**Results:**

We identified 43 patients with pathogenic *WT1* variants (truncating, *n* = 19; missense, *n* = 13; splice-site, *n* = 7; and deletions, *n* = 4). Wilms tumor was the only clinical manifestation in 10 patients, of whom 9 were female. Wilms tumor occurred in 18 of 19 patients with truncating variants, in 4 of 4 patients with deletions, and was rarer in patients with missense or splice-site variants. All patients with missense and 6 of 7 with splice-site variants developed chronic kidney disease (CKD) versus 5 of 19 patients with truncating variants (3 in adulthood, with kidney failure at the age of 24, 26, and 41 years) and 1 of 4 with a deletion. Urogenital malformations occurred predominantly in 46,XY individuals.

**Conclusion:**

Among patients with *WT1* variants, a genotype-phenotype correlation was observed for Wilms tumor risk and age of CKD onset. Although childhood-onset CKD was more common in patients with missense variants in the DNA-binding/Zinc-finger domain, other patients may develop CKD and kidney failure later in life. Therefore, life-long surveillance of kidney function is recommended. Being alert about *WT1* variants is especially important for girls with Wilms tumor who often miss additional phenotypes.

## Graphical abstract

**Figure:**
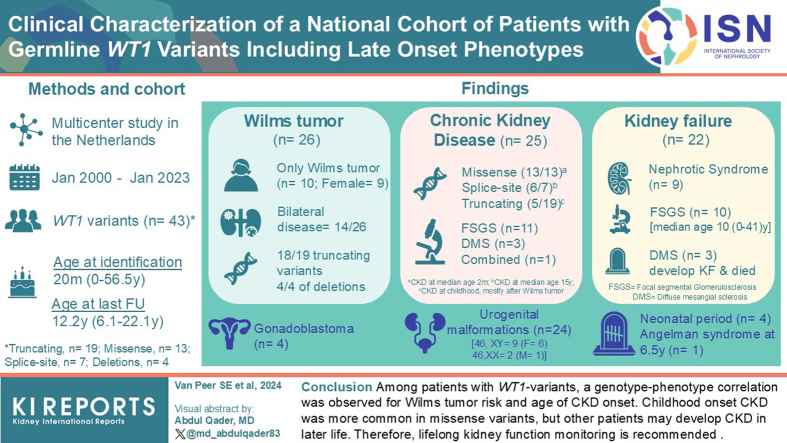

Alterations in the Wilms Tumor 1 (*WT1*) gene are among the most frequent contributors to Wilms tumor predisposition.^1^, ^2^, ^3^ *WT1* encodes a transcription factor that was first discovered as a tumor suppressor gene and was associated with Wilms tumor development in 1990.^4^ A variety of additional phenotypes have been linked to germline *WT1* aberrations, including glomerulopathy, urogenital anomalies, and more rarely gonadoblastoma.^5^^,^^6^ Previously, these phenotypes were mainly attributed to missense variants in the DNA-binding/Zinc-finger domain (exon 8 and 9, Denys-Drash syndrome) and intron 9 (Frasier syndrome) of the *WT1* gene, which was therefore referred to as the “hotspot” region. Patients with germline *WT1* variants were typically identified by the presence of early-onset glomerulopathy, and *WT1* testing was often limited to the hotspot region. However, it is now becoming clear that Wilms tumor development can be the initial presentation of a germline *WT1* variant. Many *WT1* variants that are identified in patients with Wilms tumor only are truncating variants located outside the hotspot region, in contrast to the variants that were identified earlier in patients with Denys-Drash syndrome or severe, early-onset glomerulopathy. However, testing for these variants outside the hotspot domain started years later in most genetic laboratories. Therefore, for these patients with atypical clinical presentations and/or variants outside the hotspot domain, long-term follow-up data, including the risk of developing later-onset glomerulopathy, are lacking.^2^^,^^3^^,^^7^, ^8^, ^9^, ^10^ Knowledge regarding risk for specific *WT1*-associated phenotypes, especially kidney disease, may have relevant clinical implications during and after treatment for Wilms tumor in childhood.^10^^,^^11^ Therefore, we collected genetic and clinical data from a national cohort of patients with (likely) pathogenic germline *WT1* variants and performed a genotype-phenotype analysis.

### Methods

We approached all genetic diagnostic laboratories in the Netherlands (*n* = 7) and requested pseudonymized genetic and clinical information for all patients with likely pathogenic or pathogenic germline variants in the *WT1* gene, diagnosed from January 1, 2000, until January 1, 2023. Patients were tested based on a clinical suspicion of an underlying genetic cause of their phenotype. All patients with a (likely) pathogenic germline *WT1* variant were included in this study, patients were not selected for a specific *WT1*-related phenotype. From 2005, most labs tested for the entire *WT1* gene. The molecular geneticists involved the clinicians who had requested *WT1* analysis to complete a questionnaire (Supplementary Document S1). Patients with (likely) benign variants or variants of uncertain significance were excluded.

The genetic information included the specific variant in *WT1* and, if available, patient’s karyotype. The detection method for copy number variants is specified in Supplementary Table S1. Variants were split up in truncating variants (nonsense variants and frameshift variants, introducing a premature stop codon), missense variants, exon/whole gene deletions and splice-site variants. Splice-site variants in this study are categorized into intron 9 splice-site variants and splice-site variants in other introns. Intron 9 splice-site variants induce an unbalanced production of WT1 isoforms with and without the KTS amino acids at the end of exon 9, normally expressed in a 2:1 +KTS: −KTS ratio in the kidney. Splice-site variants in other introns produce an aberrant (nonfunctional) WT1 protein, resulting in different downstream effects than intron 9 splice-site variants.^12^ Variant annotation was based on transcript NM_024426.6 of the *WT1* gene. Clinical information included sex, age, whether the patient had a Wilms tumor (including detailed information regarding histology and laterality), presence of CKD (including histology), development of kidney failure, presence of other malignancies, genitourinary anomalies, and the option to provide other phenotypic information.

The probability of absence of kidney failure was estimated using Kaplan-Meier analysis. An event was defined as having kidney failure (needing renal replacement therapy). Survival analysis was performed using R version 4.3.0^13^ and the survival package (version 3.5–7).^14^

This study was reviewed by the ethical review board of the University Medical Center Utrecht (registered under number 18/733) and the requirement for informed consent was waived. The pseudonymized genetic and clinical information of all patients was collected in a database for analysis.

## Results

The genetic laboratories in the Netherlands together identified 64 patients who tested positive for a (likely) pathogenic variant in *WT1*. We received clinical information on 43 of these patients who were all included in this study (Supplementary Table S2). The median age of patients at identification of the germline *WT1* variant was 20 months (range: birth–56.5 years). The median age at last follow-up of all patients was 12.2 years (interquartile range: 6.1–22.1 years). Further patient and variant characteristics are presented in Table 1.

**Table 1:** Characteristics of study cohort

Characteristic	Number of patients
Sex	43
Phenotypic male	16
Phenotypic female	27
Karyotype	
46,XY	13
46,XX	17
NA	13
Variant type	
Nonsense variant	13
Frameshift variant	6
Missense variant	13
Splice-site variant (intron 9)	4
Splice-site variant (other introns)	3
Deletion of 1 or multiple exons	4
Phenotype	
Wilms tumor	26
CKD	25
Kidney failure	22
Urogenital anomalies	24
Male-to-female sex reversal	5
Female-to-male sex reversal	1
Gonadoblastoma	4
Dysgerminoma	1
ITGCN	1

CKD, chronic kidney disease; ITGCN, intratubular germ cell neoplasia; NA, not available.

### *WT1* Variants

**Figure 1:**
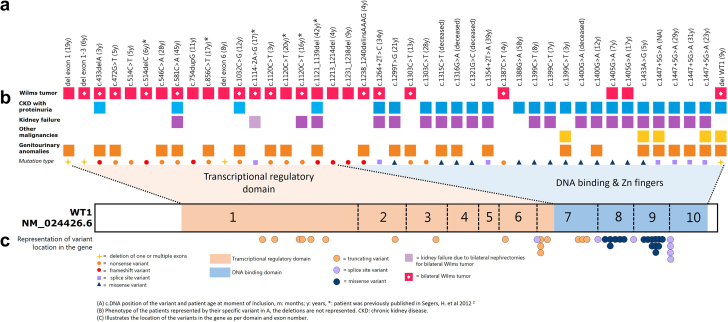
Representation of variants according to location in the *WT1* gene matched to phenotype (*WT1* transcript NM_024426). (a) cDNA position of the variant and patient age at moment of inclusion. m, months; y, years; ∗patient was previously published in Segers, H. *et al 2012*.^2^ (b) Phenotype of the patients represented by their specific variant in (a), the deletions are not represented. CKD, chronic kidney disease. (c) Illustrates the location of the variants in the gene as per domain and exon number.

**Figure 2:**
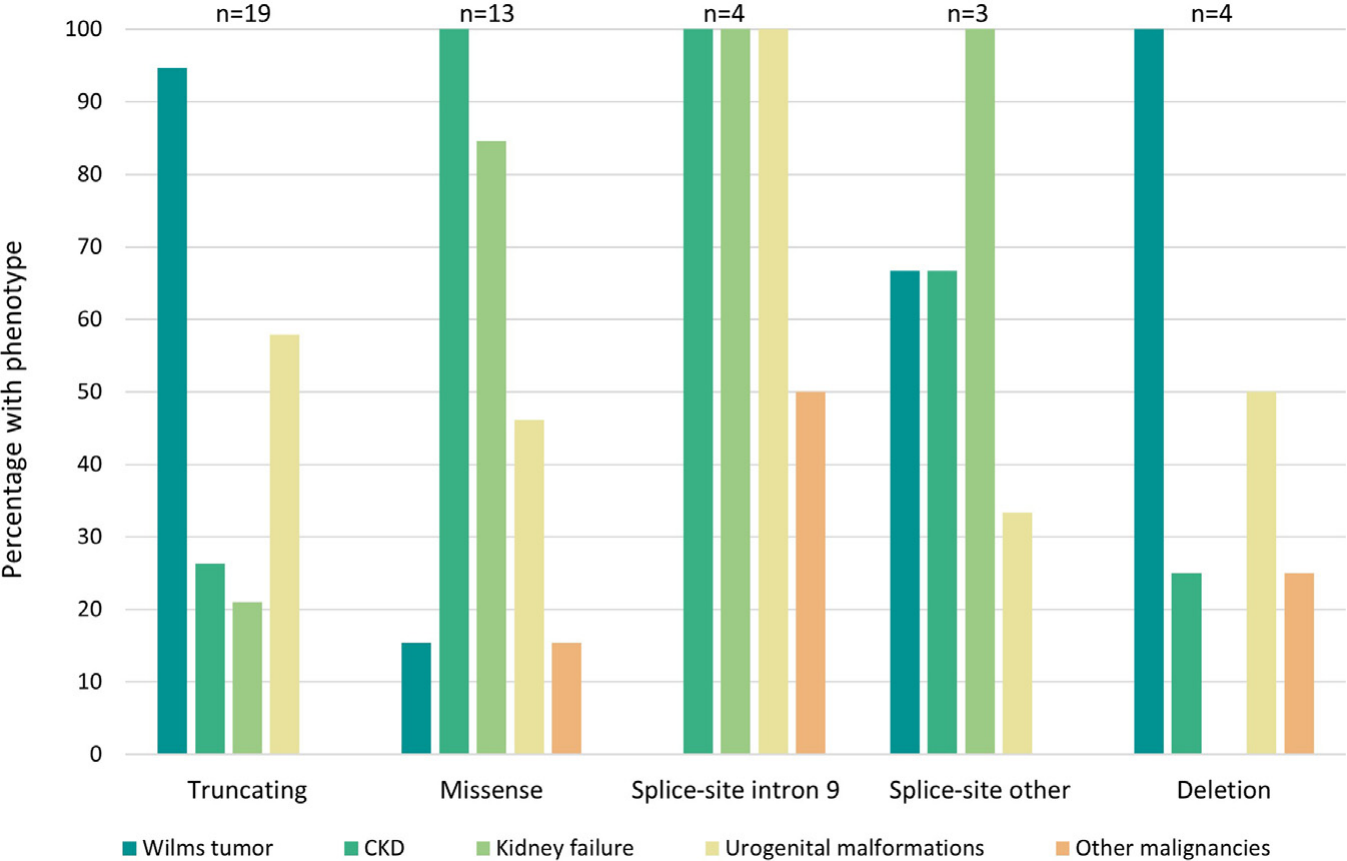
Proportion of patients with a given phenotype, according to genetic variant type. CKD, chronic kidney disease.

In total, we identified 33 unique variants in the *WT1* gene in these 43 patients (Figure 1). These included 19 truncating, 13 missense, and 7 splice-site variants (4 are located in intron 9, and 3 in other introns [intron 6, 7, and 8]). Four patients had a deletion of 1 or multiple exons of the gene. The contribution of the different *WT1* variant types to each phenotype is represented in Figure 2.

### Wilms Tumor

Wilms tumors were reported in 26 of 43 patients (60%), with a median age at (first) diagnosis of 12 months (range: 3–5.9 years). Wilms tumor was the only clinical manifestation in 10 patients (23%), of whom 9 were girls, and the first clinical manifestation in 13 patients (30%), of whom 12 were girls. Wilms tumor was reported in 18 of 19 patients with truncating variants, all with deletions, 2 of 13 with missense variants, and 2 of 7 with splice-site variants (not in patients with intron 9 variants). Five patients (4 with a missense and 1 with a splice-site *WT1* variant) underwent bilateral nephrectomies at ages < 7 years, due to untreatable kidney disease with hypertension and could therefore not develop Wilms tumors.

Fourteen patients (54% of all Wilms tumor patients) had bilateral disease (including 2 patients with unilateral Wilms tumor and contralateral nephroblastomatosis). Of 35 Wilms tumors (mostly pretreated with chemotherapy) in all 26 patients, 19 were of stromal histology, 8 were mixed type, 1 was regressive histology, and histology was unknown for 7 tumors. Nephrogenic rests were present in 13 kidneys (9 patients).

### CKD

CKD was reported in 25 of 43 patients (58%). All patients with missense variants developed CKD, at a median age of 2 months. All patients with intron 9 splice-site variants developed CKD, at a median age of 15 years, and 2 of 3 patients with other splice-site variants developed CKD, at a median age of 6 months. CKD was reported in 5 of 19 patients with truncating variants (2 with childhood-onset aged 5 and 7 years), and in 4 of 5 after treatment for Wilms tumor.

CKD with underlying focal segmental glomerulosclerosis (FSGS) was diagnosed in 11 patients by histological examination at a median age of 4 years (range: 0–22 years). Three patients were diagnosed with diffuse mesangial sclerosis (DMS) on histological examination (1 combined with FSGS), all in the first week after birth. CKD with proteinuria without histological subtype was reported for 10 patients and kidney cysts in 3 patients.

Nephrotic syndrome was reported in 10 patients, 1 patient had underlying combined DMS and FSGS (the other 2 patients with DMS died before they could develop nephrotic syndrome), 4 patients had underlying FSGS, and the histological diagnosis was not available for the other 5 patients.

Six patients who eventually developed CKD also had a history of Wilms tumor, of which 5 were bilateral. Two additional patients had already been diagnosed with CKD before the moment of Wilms tumor diagnosis. One patient underwent bilateral nephrectomies for bilateral Wilms tumors aged 1.5 years. Consequently, it was not possible to assess the contribution of the *WT1*-associated predisposition on CKD after transplantation.

### Kidney Failure

**Figure 3:**
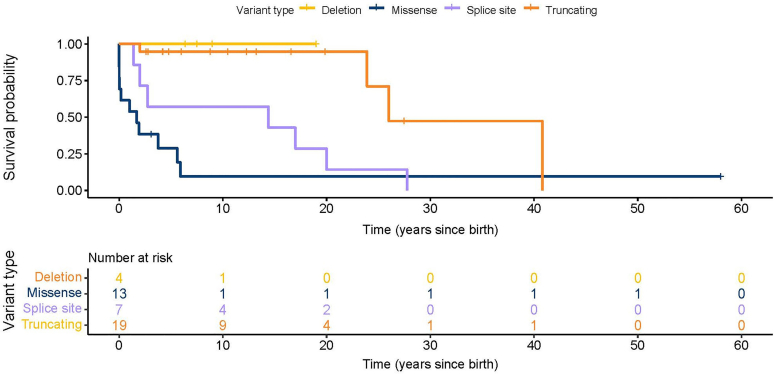
Survival analysis of kidney function.

Twenty-two patients (51%) developed kidney failure requiring dialysis or kidney transplantation, 1 patient after bilateral nephrectomy for Wilms tumor. A survival analysis was performed for the absence of kidney failure at last follow-up, specified per variant type (Figure 3).

All 10 patients with confirmed FSGS developed kidney failure at a median age of 10 years (range: 0–41 years), 3 of those had Wilms tumor in the past. The 3 patients with DMS all developed kidney failure and died, including 1 unexplained prenatal death. Nine of 10 patients who had nephrotic syndrome developed kidney failure, at a median age of 23 months (range: birth–24 years). The only patient with nephrotic syndrome who did not develop kidney failure at the time of inclusion, showed signs of slowly deteriorating kidney function at last follow-up.

Kidney failure occurred in 7 patients who had suffered from Wilms tumor in their childhood. One patient had bilateral nephrectomies for Wilms tumor and 1 patient who had kidney failure at 2 years of age had bilateral Wilms tumor at the age of 16 months (without reported surgery details). Two patients were diagnosed with CKD before developing Wilms tumor (aged 3 and 14 months) and they developed kidney failure at the age of 20 and 23 months, respectively. Another patient was diagnosed with Wilms tumor at 4 years of age and developed kidney failure following a histological diagnosis of FSGS at the age of 40 years. The sixth patient had metachronous bilateral Wilms tumor at the age of 11 months and 3 years and developed kidney failure at the age of 26 years (no histological diagnosis reported). The last patient had Wilms tumor at the age of 3 months, was diagnosed with FSGS at the age of 1 year, and developed kidney failure at the age of 28 years.

### Urogenital Malformations and Disorders of Sex Development

Twenty-four patients had congenital urogenital malformations and/or disorders of sex development. Five patients with a 46,XY karyotype had a female phenotype and 1 patient with a 46,XX karyotype had a male phenotype. Other reported urogenital malformations were cryptorchidism (*n* = 13), hypospadias (*n* = 9), kidney malformations (*n* = 2), uterine or cervical malformations (*n* = 2), hydrosalpinx or ovarian cysts (*n* = 2), ambiguous genitals without sex reversal (*n* = 3), azoospermia (*n* = 2), streak gonads (*n* = 1), 46,XX gonadal dysgenesis (*n* = 1), utricular cyst (*n* = 1) and testicular atrophy (*n* = 1) (Supplementary Table S2).

Karyotype was 46,XY in 13 patients with urogenital malformations or disorders of sex development and 46,XX in 2 patients. Karyotype was unknown in 9 patients.

### Gonadoblastoma and Other Malignancies

Gonadoblastoma was reported in 4 patients, including 1 patient (aged 23 years) with an intron 9 splice-site variant who also had a dysgerminoma, 1 patient with a *WT1* deletion at the age of 8 months, and 2 patients with missense variants in exon 9 (aged 1 and 3 years). One patient with a splice-site variant in intron 9 had a multilocular intratubular germ cell neoplasia of the testis (aged 15 years).

### Clinical Outcome

Five patients in this cohort died. One patient died shortly after birth due to cardiorespiratory distress and kidney failure caused by a combination of DMS and FSGS. Postmortem examination also revealed widely separated cranial structures, an enlarged brain, and urogenital malformations. This patient was previously published in a case report.^15^ The second patient died prenatally and appeared to have DMS-related nephrotic syndrome and sex reversal upon obduction. The third patient had nephrotic syndrome leading to kidney failure and died 5 days after birth. The fourth patient had kidney failure due to DMS, had several neurologic symptoms and died after 1 week. The last patient had Angelman syndrome (*UBE3A* germline variant) and died at the age of 6.5 years due to kidney failure and withholding of kidney replacement therapy.

## Discussion

We present genetic and clinical data from a cohort of 43 patients with germline (likely) pathogenic *WT1* variants without selection for a specific *WT1*-related phenotype, diagnosed in the Netherlands between 2000 and 2023. We observed that patients with germline *WT1* variants exhibit a spectrum of (late-onset) phenotypes, some of which can be correlated to their genotype.

Wilms tumors are among the most well-known clinical manifestations of *WT1* variants. Sixty percent of the patients in this Dutch cohort developed Wilms tumor, which is higher than in previous studies.^7^^,^^8^ These studies often selected patients based on the presence of kidney disease, which is mostly lacking in young patients with truncating *WT1* variants and Wilms tumor. This likely explains most of this difference, because in this study, patients with isolated Wilms tumor were eligible for inclusion. Patients with truncating variants in *WT1* had the highest frequency of Wilms tumor occurrence (95%) and they often had bilateral tumors (56%), as described previously.^7^^,^^10^^,^^16^ Other malignancies that may occur less frequently in patients with *WT1* variants include germ cell tumors, such as gonadoblastoma. Patients at risk for these tumors typically have a 46,XY karyotype and a disorder in gonadal (testicular) development, as confirmed by this study.^8^^,^^16^ Therefore, these patients should be closely monitored to ensure timely intervention of the tumor.

Although childhood-onset CKD was much more common in patients with missense variants, all located in the DNA-binding/Zinc-finger domain, patients with other types of *WT1* variants are at risk for CKD as well. The onset of CKD is often later in life and the progression rate is slower than in patients with missense variants,^7^^,^^8^^,^^10^^,^^11^ but can still result in kidney failure. Probably, the early onset and progressive disease in patients with missense variants are explained by a dominant negative effect of the mutant allele.^17^ Such dominant negative variants result in a mutant protein that directly or indirectly interferes with the normal protein. Therefore, not only is half of the protein function lost due to the pathogenic variant, but the wild-type protein is also not functioning normally to a certain extent.^17^, ^18^, ^19^ This often results in a more severe phenotype than variants that produce a truncated or nonfunctional protein.

Among patients with missense variants, a variety in the severity of kidney disease is observed when looking at age of onset and disease progression. Nagano *et al.*^17^ have shown that it likely matters whether a variant is in the specific DNA-binding or C2H2 sites in exon 8 and 9, both important for the DNA-binding capability of the transcription factor. Variants directly affecting the DNA-binding or C2H2 sites seem to show an earlier age of onset and faster disease progression compared to missense variants located in other amino acids in exon 8 and 9.^17^ We observed a similar trend in our own data; however, the numbers are too small to perform a statistical analysis and support this statement. In patients with truncating variants, splice-site variants (not in intron 9) or deletions, Wilms tumor is often the first clinical manifestation. Especially, patients with a 46,XX karyotype frequently lack other recognizable phenotypes such as urogenital anomalies and may therefore not be selected for *WT1* testing. However, to postpone the possible onset of *WT1*-related CKD and kidney failure, it is essential to preserve as much kidney tissue as is oncologically feasible (nephron-sparing surgery) throughout the treatment course for Wilms tumor. This highlights the significance of early awareness about underlying germline genetic conditions in patients with Wilms tumor, even though additional phenotypes are lacking. In addition, timely involvement of a pediatric nephrologist is important for patients with *WT1* variants, and life-long surveillance for CKD is recommended.

The underlying mechanism of CKD in patients with *WT1* variants is typically a progressive glomerulopathy.^8^^,^^16^ Similar to the study of Lehnhardt *et al.*,^7^ we found that DMS was only confirmed in patients with missense variants and mostly had an earlier onset than other glomerulopathy types.^7^ Patients with both types of splice-site or truncating variants more often had FSGS or unspecified histology CKD. Unfortunately, histological confirmation of glomerulopathy was missing in some patients. It is important to note that the median age of patients in this cohort is still quite low (Table 2). Therefore, the frequency of CKD may still increase, especially in patients with truncating or splice-site variants (both types), because the onset of kidney disease in these patients is often later in life.

**Table 2:** Phenotype according to type of variant

Variant type	Number. of pts	Clinical gender	Exon/intron (number. of pts)	Median age at last follow-up (yr)	Wilms tumor *n* (%)	CKD *n* (%), age of onset in yr	Kidney failure *n* (%), age of onset in yr	DSD *n* (%)	Urogenital malformation *n* (%)	Other malignancies	Mortality
Missense	13	3M, 10F	Exon 8 (4), Exon 9 (9)	8.0	2 (15), 0 bilateral	13 (100)	11 (85), range 0-6	3 (33.3)	6 (46)	2 (gonadoblastomas)	5/13
Truncating	19	10M, 9F	Exon 1 (6), Exon 2 (1), Exon 3 (1), Exon 6 (1), Exon 7 (7), Exon 8 (2), Exon 9 (1)	12.8	18 (95), 10 bilateral (56)	5 (26)	4 (21), 2, 24, 26, 40	0	11 (58)	0	0/19
Splice-site	7	2M, 5F	Intron 6 (1), Intron 7 (1), Intron 8 (1)	29.7	2 (67), 2 bilateral (100)	2 (67), 0, 1, NA	3 (100), 1^a^, 3, 28	0 (0)	1 (33)	0	0/3
			Intron 9 (4)	18.6	0	4 (100), 5, 9, 14, NA	4 (100), 2, 14, 17, 20	1 (25)	4 (100)	2 (1 gonadoblastoma and dysgerminoma and 1 ITGCN)	0/4
Deletion	4	F	*WT1* gene	9	Yes (bilateral)	CKD, cysts (NA)	No	Yes	Yes	Gonadoblastoma	0
		M	Exon 1	19	Yes	No	No	No	Yes	No	0
		F	Exon 1-3	6.4	Yes (bilateral)	No	No	No	No	No	0
		F	Exon 6	7.5	Yes	No	No	No	No	No	0

CKD, chronic kidney disease; DSD, disorder of sexual development; F, female; ITGCN, intratubular germ cell neoplasia; M, male; NA, not available; pts., patients.

a Kidney failure due to bilateral nephrectomies for Wilms tumor.

Several patients developed CKD after having been treated for Wilms tumor in the past, which is partly attributable to oncologic treatment. It was not feasible to discriminate between CKD resulting from oncologic treatment and CKD that was more likely to be caused by the *WT1* variant. However, studies describing long-term kidney follow-up after nonsyndromic Wilms tumor treatment report variable but lower percentages of impaired kidney function and kidney failure than we observed (CKD in 9/26 patients [35%] and kidney failure in 7/26 patients [27%]) in this cohort. Impaired kidney function (variably defined as albuminuria, proteinuria and/or declined glomerular filtration rate) after nonsyndromic (unilateral and bilateral) Wilms tumor treatment varies in the literature from almost negligible to approximately 20%, but mostly around 10%.^20^, ^21^, ^22^ Kidney failure varies from 1% to 2% in patients with unilateral Wilms tumors and 12% to 20% in bilateral Wilms tumor survivors.^21^^,^^23^, ^24^, ^25^ This was also confirmed by long-term follow-up studies of patients with Wilms tumor and underlying *WT1* variants, WAGR, or urogenital malformations (as a proxy for possible *WT1* involvement), varying from 36% to 90% depending on the type or location of the variant.^22^, ^23^, ^24^

In the Netherlands, the largest diagnostic laboratory for *WT1* testing started analysis of the complete gene from 2005 onward, rather than only the exon 8/9 “hotspot” region. In addition, since the national centralization of pediatric renal cancer care at the end of 2014, more children with Wilms tumor (who often have truncating variants in the non-hotspot domain of the gene) were tested for *WT1* aberrations. This has yielded more patients with variants in other domains than the hotspot domain. Both developments explain the aforementioned short follow-up time for this patient group with truncating variants. We assume that previously, truncating germline *WT1* pathogenic variants may have been missed, particularly in (46,XX) patients with Wilms tumor without additional *WT1*-characteristic features.

In line with previous cohorts, the most common urogenital anomalies included hypospadias and cryptorchidism.^7^^,^^8^^,^^10^ We and others did not find a correlation between anomalies of the urogenital tract and type or location of the *WT1* variant.^8^ Karyotype however, did correlate with the presence of urogenital malformations. Although the frequency of urogenital malformations, including disorders of sexual development, might be underestimated in this cohort due to missing information on karyotype in 13 patients, we observed that these were mostly present in patients with a 46,XY karyotype, as reported by others.^7^^,^^8^^,^^10^^,^^11^^,^^26^^,^^27^ A partial explanation could be that the WT1 protein plays a role in the differentiation of mesonephric structures during embryonal development, which is more important in male than in female genital development.^26^ However, it might be that female patients have more (underdiagnosed) abnormalities of the internal genitalia. To prevent underdiagnosis of disorders of sexual development in clinically female patients with a germline *WT1* variant, their sex chromosomes should be analyzed. This is important because sex reversal can lead to germ cell tumors and infertility, significantly affecting the patient. Interestingly, 1 patient with a 46,XX karyotype had a male phenotype (patient 36 in Supplementary Table S2), which is much rarer than 46,XY sex reversal, but has previously been documented in 10 other patients who had variants within the same region in intron 9.^28^, ^29^, ^30^, ^31^, ^32^ These variants are suggested to cause disruption of transcription of the fourth Zinc finger of the protein, leading to gonadal masculinization.^28^, ^29^, ^30^, ^31^, ^32^ In general, it is advised to perform genetic evaluation, including *WT1*, for neonates or children diagnosed with urogenital anomalies, including ambiguous genitals.

There is a phenotypic variability between patients with the same variant, both in the literature and this study. Phenotypes matching the classic syndromes, such as Denys-Drash and Frasier syndrome are rarely completely present. Instead, it rather seems that *WT1* variants cause a spectrum of phenotypes, in which an association with genotype exists but does not strictly apply. In addition, we found that phenotypes such as kidney disease and failure may not be present in childhood but may present later in life. Therefore, these data indicate that syndrome definitions need to be revised, as was already proposed.^7^

Our study is limited by the rarity of *WT1* variants which did not allow for a statistical analysis of genotype-phenotype correlations. Moreover, due to the retrospective data collection, some patients could not be included in this study, because we were dependent on response from local geneticists. This particularly applies to patients who were diagnosed earlier, introducing possible bias in the cohort toward recently diagnosed patients, who more frequently have truncating variants. Furthermore, given that copy number variants in the gene require advanced detection techniques, it is possible that such variations were previously missed. This could potentially have introduced a bias toward observing a higher prevalence of copy number variants in patients diagnosed more recently. Because patients were tested for *WT1* variants based on clinical suspicion, there is likely a bias toward including patients with phenotypes known to be associated with *WT1* variants. Consequently, this study design is not appropriate for discovering novel *WT1* phenotypes. Histological examination was not possible in all patients with kidney disease, and an estimation of the remaining kidney tissue in patients with Wilms tumor was mostly lacking, both of which would have been interesting with regard to kidney function follow-up. Nevertheless, to our knowledge, this is the largest cohort of patients with (likely) pathogenic germline *WT1* variants that is unselected for a specific phenotype.

In conclusion, we observed that patients with *WT1* variants present a spectrum of phenotypes with a genotype-phenotype correlation for the risk of Wilms tumor development and age of onset of CKD. Although childhood-onset kidney disease was much more common in patients with missense variants, other patients are at risk for CKD later in life. Therefore, life-long surveillance of kidney function is recommended for all patients with a *WT1* variant. Awareness of an underlying *WT1* variant is especially important in young females with Wilms tumor, because they often present without additional recognizable phenotype, but may still develop CKD resulting in kidney failure in the future. Because the cohort of patients who initially presented with Wilms tumor is still quite young, future follow-up studies are warranted to further characterize their risk of late-onset kidney disease.

## Disclosure

All the authors declared no competing interests.

## Supplementary Material

Supplementary File (PDF and Excel) **Document S1.** Case report file.

Table S1 Copy number variant detection method.

Table S2 All patients were included in the study.
