# Supplementary material for: Clinical Characterization of a National Cohort of Patients With Germline WT1 Variants Including Late-Onset Phenotypes
Source: Kidney Int Rep. 2024 Sep 16;9(12):3570–9. doi: 10.1016/j.ekir.2024.09.007 (PMC11652072; doi:10.1016/j.ekir.2024.09.007)
Supplement: Supplementary File (PDF and Excel) — Document S1. Case report file. [file mmc1.pdf]

SUPPLEMENTARY MATERIAL

List of contents

- Supplementary Document 1: case report file

Case:

WT1 study

GENETIC DIAGNOSIS	
**WT1 mutation**	Mutation:………………………………………………….<br><br>Age at diagnosis:…………(years, months) Current age………………….(years, months)
**MEDICAL HISTORY & CLINICAL FINDINGS**	
**Gender**	☐ Female ☐ Male **Karyotype:** ☐ 46XX ☐ 46XY
**Wilms tumor**	☐ No ☐ Yes ☐ Unknown<br><br>**If yes:**<br>Age at diagnosis: ……………………………………………………… (years, months)
	**Tumor characteristics:**<br>☐ Unilateral ☐ Bilateral<br><br>**Metastasis?**<br>☐ Yes ☐ No ☐ Unknown<br><br>**Nephrogenic rests? (left kidney)**<br>☐ Yes ☐ No ☐ Unknown<br><br>**Nephrogenic rests? (right kidney)**<br>☐ Yes ☐ No ☐ Unknown<br><br>**Multifocality? (left tumor)**<br>☐ Solitary ☐ Multifocal ☐ Unknown<br><br>**Multifocality? (right tumor)**<br>☐ Solitary ☐ Multifocal ☐ Unknown<br><br>**Histology (left tumor):**<br>*Specify and/or add a copy of pathology report (anonymized)*<br>……………………………………………………………<br>……………………………………………………………<br>……………………………………………………………<br><br>**Histology (right tumor):**<br>*Specify and/or add a copy of pathology report (anonymized)*<br>……………………………………………………………<br>……………………………………………………………<br>……………………………………………………………
**Other malignancies/ tumors?**	☐ No ☐ Yes ☐ Unknown<br><br>If yes, specify: ………………………………………………………………...…<br><br>Age at diagnosis: ……………………………………………………(year, months)
**Genitoury anomalies**	☐ No ☐ Yes ☐ Unknown<br>**If yes, specify:**<br>☐ Hypospadias<br>☐ Cryptorchidism<br>☐ Streak gonads<br>☐ Ambiguous genitalia<br>☐ Other, specify:……………………………………………...<br>……………………………………………………………………
**Kidney disease/ renal failure?**	**Kidney disease:**<br>☐ Proteinuria ☐ Nephropathy* ☐ Unknown<br>Age at diagnosis (chronic) kidney disease: ……………………………….. (years, months)<br>* **If nephropathy, specify:** ……………………………………………………………………………..<br>……………………………………………………………………………………………………...<br><br>**Renal failure with an indication for dialysis or renal transplant?**<br>☐ No ☐ Yes ☐ Unknown<br>**If yes:**<br>Age at diagnosis end-stage renal disease:……... (years, months)
**Other phenotype present?**	☐ No ☐ Yes ☐ Unknown<br><br>**If yes:**<br>Specify additional findings: ………………………………………..…………………………<br><br>………………………………………….………………………………………………………..
